# Refinement of the Well-being in Pregnancy (WiP) questionnaire: cognitive interviews with women and healthcare professionals and a validation survey

**DOI:** 10.1186/s12884-022-04626-x

**Published:** 2022-04-15

**Authors:** Laura Kelly, Research Officer, Jennifer J. Kurinczuk, Ray Fitzpatrick, Fiona Alderdice

**Affiliations:** 1 Health Services Research Unit, Nuffield Department of Population Health, University of Oxford, Oxford, UK; 2 Harris Manchester College, University of Oxford, Oxford, UK; 3 NIHR Policy Research Unit Maternal and Neonatal Health and Care, National Perinatal Epidemiology Unit, Nuffield Department of Population Health, University of Oxford, Richard Doll Building, Old Road Campus, Headington, Oxford, OX3 7LF UK

**Keywords:** maternity care, patient-reported outcomes, questionnaire, chronic conditions, pregnancy, well-being

## Abstract

**Background:**

Measuring positive and negative aspects of well-being during pregnancy and childbirth is important for both healthy women and women who are living with long-term health conditions (LTCs). This study aimed to further refine the Well-being in Pregnancy (WiP) questionnaire and to incorporate LTC specific items where appropriate.

**Methods:**

A multi-method study. Cognitive interviews with pregnant or postpartum women (*n* = 11) and consultations with healthcare professionals (*n* = 11) and public representatives (*n* = 4) were conducted to explore the acceptability of existing WiP items and content. Items were refined and subsequently administered on an online survey (*n* = 768). Item reduction steps and exploratory factor analysis were performed on survey data. Convergent validity was examined using Pearson correlation coefficients to compare relationships with other included validated assessments.

**Results:**

Following amendments to three items, the addition of eight core WiP items and five LTC specific items, a total of 25 items were considered relevant and appropriate for use with pregnant women. Analysis of survey data reduced the questionnaire to 12 items measuring three core WiP scales; 1) Concerns over support after birth, 2) Positive pregnancy and, 3) Confidence about motherhood, and a five item standalone LTC specific scale. All scales demonstrated good validity and internal reliability. Scores for the three core scales moderately correlated with established well-being measures indicating that they were measuring similar, yet distinct concepts.

**Conclusions:**

Analyses confirmed good psychometric properties of the refined WiP questionnaire. The use of pregnancy specific well-being measures, such as the WiP, provide a route into asking women in more detail about how their care may be tailored to support them and also facilitates positive conversations with women about how care and experience of pregnancy and childbirth may be enhanced further.

**Supplementary Information:**

The online version contains supplementary material available at 10.1186/s12884-022-04626-x.

## Background

Subjective well-being is a multi-faceted concept that encompasses both positive and negative emotions, and an evaluation of satisfaction with life and psychological functioning [1]. While research into subjective well-being has grown significantly in recent years, it remains poorly defined [2]. The term well-being is often used interchangeably with happiness, flourishing and quality of life. There is also much debate about what constitutes subjective wellbeing. The Wellbeing in Pregnancy measure builds on Diener’s model [3] which recognises the multi-dimensionality of subjective wellbeing and highlights the inclusion of positive and negative affect and a cognitive components [4]. The affective component (often referred to hedonic wellbeing) needs to include both positive affect and negative affect in a full assessment of well-being [5]. The cognitive component may be viewed differently depending on philosophical perspective as Life Satisfaction (evaluative component) or eudemonia (meaning in life) [6]. As the cognitive component reflects the conditions and circumstances of life as a whole, additional measurement of domain satisfaction represents a focused evaluation of some specific aspect of one’s life e.g. income or partner satisfaction [6].

A recent systematic review of the impact of pregnancy on subjective wellbeing found life satisfaction, happiness, and mental component of quality of life, were found to be high during pregnancy, but positive emotion and physical components of quality of life had decreased [7]. A number of generic well-being measures are widely used, for example, the Satisfaction with Life Scale, WHO5 and Positive and Negative Affect Scale [8–10], and have more recently being used in perinatal research. Research to date suggests that pregnant women have higher levels of subjective well-being than mothers of young children (age 0–2 years). Both mothers and pregnant women were more likely to report that they were happy and felt like the things they do were more meaningful than other women [11]. A small number of studies have found high subjective well-being during pregnancy to be associated with a number of positive outcomes including a reduction in the risk of preterm birth [12], better feeding practices [13] and possible protection against postnatal depression [14]. In addition, a systematic review by Rasmussen et al. (2009) found women who were more optimistic during pregnancy were more likely to have had fewer miscarriages and babies with a healthy birthweight [15].

In the broader wellbeing field, the link between physical health and subjective wellbeing is increasingly recognised. For example, at the physiological level, positive emotions have been found to improve immune, cardiovascular, and endocrine functioning. In contrast, negative emotions are detrimental to these processes [16]. This is also reflected in the perinatal period with women who rate their own health as poor or have a longstanding disability or illness have lower levels of well-being, and women who have experienced complications with their pregnancy or birth are less likely to feel good about themselves or to be satisfied with their lives [11, 17]. This highlights the need to be aware of and explore the impact of long term health conditions on wellbeing during pregnancy.

While generic well-being measures are increasingly being used, research suggests additional benefit in having domain specific measures. Domain satisfaction and life satisfaction are generally highly correlated but they can diverge therefore, measurement of domain satisfaction allows the examination of variations in well-being related to specific circumstances [18]. As pregnancy is a major life event that could impact on other aspects of life including health and relationships, a domain-specific well-being measure would help identify the unique contribution of pregnancy specific subjective well-being and identify important areas of maternal need. This has been demonstrated in pregnancy specific measures of negative affect which have shown more predictive value than nonpregnancy specific measures [19, 20]. In addition, the majority of women using maternity services are healthy, therefore applying a well-being perspective during pregnancy, which focuses not just on negative but also on positive changes, may reduce the risk of over-pathologising emotional changes [21]. Complementing this perspective on positive and negative aspects of well-being during pregnancy with a domain specific well-being measure that can evaluate the impact of LTCs on well-being during pregnancy will provide valuable data to inform policy and practice.

The Well-being in Pregnancy Scale (WiP) was developed to fill this measurement gap. This 12 item measure, which was previously developed following focus groups with women who were pregnant and women who had recently given birth, demonstrates good psychometric properties and has shown significant correlations with other general well-being measures [1]. As part of the ongoing development of the WiP, we explored the value of using the WiP among pregnant and postpartum women and healthcare professionals working within maternity services within the UK. This study therefore aimed to assess and refine the modified core WiP items, add an additional WiP LTC module where necessary, and carry out a psychometric validation of the refined item content.

## Methods

### Design and ethics

**Fig. 1:**
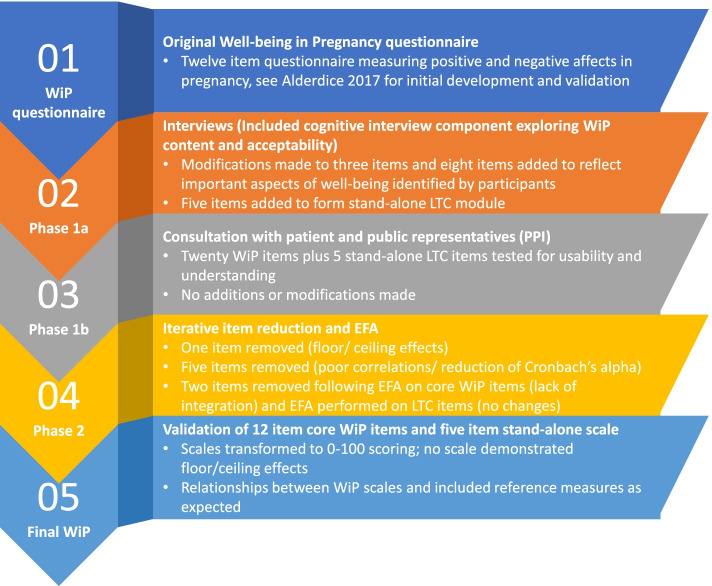
Sequence of steps taken to refine and validation the Well-being in Pregnancy questionnaire

A multi-method study consisting of two phases. Phase 1 aimed to confirm the suitability of existing WiP items and make modifications or add items where necessary. Phase 2 aimed to carry out a psychometric validation of the refined item content using appropriate quantitative methods. Study design was developed in line with internationally recognised standards for patient reported instrument development, such as that promoted by the US Food and Drug Administration (FDA) and the European Medical Agency (EMA) [22, 23]. Figure 1 shoes the sequence of iterative steps taken to refine and develop the WiP questionnaire.

Ethical approval was granted by the University of Oxford’s Medical Sciences Interdivisional Research Ethics Committee (Reference Number: R61498/RE001). For Phase 1, women and healthcare professionals taking part in qualitative interviews provided informed consent through signing an online consent form. For Phase 2, women were asked to confirm their eligibility to take part in the study through confirming they are aged 18 or over, that they are currently pregnant and that they agreed to take part. Consent was assumed upon anonymous survey submission.

### Phase 1a: Cognitive interviews with women and healthcare professionals

Qualitative interviews took place with women (*n* = 11) and healthcare professionals (*n* = 11) who were taking part in a wider study exploring the acceptability and feasibility of using health and well-being measures among pregnant and recently pregnant women who were living with LTCs [24]. Part 2 of the qualitative interviews took place in the form of a cognitive interview which aimed to assess the 12 items within the WiP questionnaire. During the cognitive interview, items were tested for relevance, understanding and possible gaps in content when measuring well-being in pregnancy. Participants were asked probing questions to confirm their understanding of the items and each item’s relevance to well-being in pregnancy [25, 26]. In cases where items appeared ambiguous or sensitive, items were amended. Postpartum women were asked to provide a retrospective view on well-being during their most recent pregnancy. See Additional file 1 for interview guide.

#### Study participants and procedure

Participants were women and healthcare professionals taking part in a wider study to explore the feasibility of using health and well-being measures in women living with LTCs [24]. On agreeing to take part, women were given the opportunity to ask any questions about the research and asked to complete an online consent form. In addition to the information provided on the participant information sheet, LK gave a verbal explanation as to why the research was being conducted and an outline of her personal role in the research before the commencing interviews. Participants were given a GBP £30 voucher for their participation. Women were required to be living in the UK, be at least 18 years old and either currently pregnant or had given birth within the past year to take part. Healthcare professionals were required to have experience of caring for pregnant or postpartum women in connection to their LTC (such as, GPs, midwives, obstetricians and health visitors). See [24] for further details.

Interviews took place over the phone, were recorded and used the verbal probing method which allowed women to provide uninterrupted feedback followed by a focussed interview to gain a deeper understanding of responses [27, 28]. Interviews were conducted by a trained qualitative researcher, LK, who had no contact or relationship to participants prior to the research taking place. Interview transcription was outsourced, and accuracy checked by LK on their return. QSR NVIVO 11 software was used identify comments relating to specific items (for example, where a participant expanded on their interpretation of an item) and codes were generated to allow for the identification of any additional topics or concepts not already incorporated within the existing WiP items. Participant comments were summarized according to each questionnaire item and collated within an Excel document (Microsoft) by LK. This allowed within-case (how the item fits within the questionnaire as a whole) and between-case (interpretation of items across the sample) analysis [28]. Interpretation difficulties or inconsistencies were discussed among authors and amended where appropriate. Analysis of the Excel document by the research team also ensured all that all questionnaire items had been adequately discussed and probed with each participant. The Excel document was completed concurrently with the interviews taking place allowing the research team to identify when no new comments were arising from both the women and healthcare professionals taking part.

### Phase 1b: Consultation with patient and public representatives (PPI)

The modified WiP was pretested among PPI representatives for ease of completion and understanding. Women who had previously taken part in Phase 1a interviews, and had consented to be contacted again for the purposes of PPI engagement, were emailed and asked if they would like to provide feedback on the amended questionnaire and given a GBP £30 voucher for their time.

Consultations took place over the phone and women were given the opportunity to provide uninterrupted feedback regarding the questionnaire items followed by a focused discussion to gain a better understanding of responses. Probing questions were used to confirm understanding of the amended items and confirm their relevance to well-being in pregnancy.

### Phase 2: Psychometric Validation

A web-based survey, formatted using Qualtrics survey software, was developed for administration to women who were currently pregnant. Initial screening questions were designed to determine whether a respondent had one or more LTC. Women who did not specify having a LTC were directed to Survey, Section 1 and women who indicated having one or more LTC were directed to Survey, Section 2. Section 1 included the following assessments: the modified WiP (core items only), the EQ-5D-5L, the Satisfaction With Life Scale (SWLS), the WHO-5 Well-being Questionnaire and, the Positive and Negative Affect Schedule (PANAS). Section 2 included the modified WiP core items with the LTC specific five item module, the EQ-5D-5L and the Long-term Conditions Questionnaire (LTCQ). To minimise responder burden, Section 2 for women living with LTCs did not include the three general well-being questionnaires included in Section 1. All assessments are outlined in further detail below. All women were asked demographic questions and questions relating to experiences of maternity care. The minimum sample size sought followed recommendations of ten responders per questionnaire item [29, 30]. Given the length of the revised questionnaire, 250 women were therefore required for the LTC population (25 items) and a minimum of 200 women were sought for the healthy population (20 items).

#### Study participants and procedure

All women were required to be living in the UK, be at least 18 years old and currently pregnant to take part in the online survey. Women who were not currently pregnant were excluded from taking part. Women were recruited through paid social media (Facebook and Instagram) advertisements. In addition, women living with a LTC were recruited through research advertisements posted on relevant mother and baby and condition specific organisations websites/ their associated social media. Examples of organisations include; Asthma UK, Guts UK, Epilepsy Action, Diabetes UK, Thyroid UK, National Maternity Voices, Mums Like Us and Netmums. Women were also contacted through the NPEU’s extensive public and public involvement and engagement group. Research advertisements were posted with a link to the information sheet and questionnaire link.

#### Assessments

The Satisfaction With Life Scale (SWLS) contains five items, developed to assess satisfaction with the respondent’s life as a whole using a seven point scale from ‘strongly agree’ to ‘strongly disagree’ [8]. Possible scores range from 5 to 35, with a score of 20 representing a neutral point on the scale. Scores 5–9 indicate the respondent is extremely dissatisfied with life, scores between 31 and 35 indicate the respondent is extremely satisfied with life.

The WHO-5 Well-being Questionnaire is a five item scale measuring positive psychological well-being within the past 2 weeks using a 6 point scale ranging from ‘all of the time’ to ‘at no time’ [9]. Total raw scores range from 0 to 25. Scores are multiplied by 4 resulting in a final score where 0 represents the worst imaginable well-being and 100 represents the best imaginable well-being.

The Positive and Negative Affect Schedule (PANAS) is a 20-item scale used to assess positive and negative affect [10]. All the items are rated on a scale ranging from ‘very slightly or not at all’ to ‘extremely’. The ten positive items are summed and the ten negative items are summed, resulting in a score range of 10–50 for each scale.

The EQ-5D-5L is a generic health status measure containing five questions, each on one domain (mobility, self-care, usual activities, pain and depression/anxiety), and a Visual Analogue Scale (VAS) [31]. Each question has five response options, and the scores of the five domains can be transformed into a single index value. The score typically ranges from 0 (death) to 1 (best possible health), although a small number of scores can be obtained to indicate health states worse than death. The VAS ranges from 0 (worst imaginable health state) to 100 (best imaginable health state).

The Long-Term Conditions Questionnaire (LTCQ) measures the impacts of living with mental and/or physical long term conditions [32]. It contains 20 items and is suitable for use in both health and social care settings. LTCQ item responses range from Never-Always and are scored on a scale from 0 (most negative response) to 4 (most positive response). All 20 items are scored as a single composite measure.

Permissions to use licenced measures were acquired where necessary (EQ-5D-5L and LTCQ).

#### Analysis

All analysis took place with SPSS (version 27) statistical software package [33]. Demographic data were presented using descriptive statistics. WiP items were recoded so that 0 = low levels of well-being and 3 = high level of well-being. Items then underwent a number of item reduction steps, including checks for the presence of high floor and/ or ceiling effects, the presence of items which demonstrated large numbers of weak correlations with other items and, the presence of items which reduced the internal reliability of the overall measure. Floor and ceiling effects were defined as < 5 and > 40% of respondents endorsing the most negative and positive response options, respectively [34]. An item correlation matrix was used to identify items demonstrating poor correlations (< 0.2) with a large number of items. Poor correlations with a large number of items can indicate a particular item is measuring a similar construct to other items in the scale (i.e. they do not share variance). Low item-to-total correlations (< 0.3) and items which lowered the Cronbach alpha value were identified through reliability analysis.

To ensure suitability of exploratory factor analysis (EFA) on the data set, tests including Bartlett’s Test of Sphericity (*p* < 0.05) [35] and Kaiser-Meyer-Olkin (KMO) value (recommended value of 0.6) [36] were carried out. Following EFA, factors demonstrating an Eigenvalue of greater than 1 were extracted and rotated using an oblique (Direct Oblimin) rotation allowing correlation between factors [37, 38]. While both the Structure and Pattern matrices were used in interpreting output, and the Structure matrix offered primary guidance for interpretation [35].

On identification of sub-scales present, scales were transformed to a 0–100 score and examined for floor and ceiling effects (considered more than 20% of responses scoring 0 or 100). Population characteristics were examined to explore any potential covariate factors. Convergent validity was examined for the newly developed scales through assessing Pearson correlation coefficients (r) to compare relationships with the other validated assessments included. It was hypothesised that the satisfaction and well-being instruments (SWLS, the WHO-5 and the PANAS) would correlate moderately, the EQ-5D-5L would have weak to moderate correlations with the newly developed sub-scales. Finally, it was predicted that the LTC specific module would have moderate to strong correlations with LTCQ scores. Internal consistency of the newly developed scales was assessed with the Cronbach alpha statistic (> 0.7) [39].

## Results

### Phase 1a: Cognitive interviews with women and healthcare professionals

Eleven women with pre-existing LTCs and 11 healthcare professionals took part in cognitive interviews. Women were a mean age of 32.9 years (SD 3.8, range 26–38 years). At the time of interviewing, five women were pregnant and six were postpartum. Healthcare professional’s taking part included a GP with a special interest in perinatal health and high-risk pregnancies, a specialist midwife for hypertension and renal disease, a diabetes specialist midwife, a midwife and infant feeding co-ordinator, three obstetricians, an obstetric physician, a consultant perinatal psychiatrist and two health visitors. See [24] for further details.

Following feedback on WiP items and content during the cognitive telephone interviews, modifications were made to three items and a further eight items were added to reflect further areas of well-being during pregnancy which were considered important by those interviewed (Table 1). The amendments to three existing items made subtle changes to wording so that women would be able to feel more comfortable in their responses. For example, ‘I feel I have bonded with my baby’ became ‘I feel I connected to my baby’ following feedback from both women and healthcare professionals advising that more subtle wording may allow women to communicate their worries more freely without raising anxiety that they should feel an instant bond with their baby. One woman said:

*That’s [WiP bonding item] quite a hard one because if you're further on obviously you’ve got a bump, you can feel it moving, feel it kicking. Wereas, early on it’s still quite surreal and … without having that first scan you almost are a bit more reserved … You want to protect your emotions a little but more so you could … By answering that you could then feel, I don’t know, a bit of guilt by being like, ‘Oh God, I haven’t bonded with my baby yet, I feel bad for that,’ when actually that’s probably quite normal because you're so early on.* W7, Asthma, Chronic rhinitis

Wording was also amended to address concerns regarding items relating to women’s satisfaction with care. Healthcare professionals in particular were concerned that a woman currently in their care would feel unable to express negative views on care received. Amendments were therefore made to make the item reflect overall care received and not care specific to any one healthcare professional. Two healthcare professionals explained:

*… if you're a woman...and you're about to see the midwife, your question twelve, “I feel supported by the health professionals involved in my care during my pregnancy”. I mean are you going to talk... are you going to say, “Not at all?”* SH2, General Practitioner

*Because if you're a midwife that’s been caring for them for the whole way through pregnancy and then they want to say it is actually a negative experience … a little bit of a concern as to whether or not you might not get a truthful answer on that...* SH11, Health visitor

Whilst these views were less common among women interviewed, one did express concern that responses may impact on care received:

*I think I'm just a little bit cautious about questionnaires that you complete that are just fed back instantly, um and then alters your care … .it's not very anonymous to provide that information … how honest people can be on these questionnaires if then it’s fed back into their care?* W6, Ulcerative colitis

During the interviews, women and healthcare professionals were asked to reflect on potential gaps within the content of the questionnaire. Although the measure was designed to focus on well-being during pregnancy, it was clear that many women were concerned during their pregnancy about life after birth and how they will adjust to motherhood. A number of women said:

*… there’s nothing about … how you feel about your life afterwards, and I think during pregnancy you’re worried about that, or at least I was... especially with a first child … You can’t really understand how that’s going to impact your life until it happens, so there is a certain amount of anxiety about what your life’s going to be like afterwards that impacts you during pregnancy.* W12, Endometriosis, underactive thyroid

*… .[the WiP items are] talking about during the pregnancy, [what is missing is] … stuff to do with post-pregnancy...Like, “... do you have any concerns?”* W2, Diabetes (Type 1), hypothyroidism

*… maybe include something about, you know, like baby blues and things … it can get really bad. One of my friends has experienced it really bad, so like... Or postnatal depression … And the support available for it.* W5, Asthma, Hypothyroidism

An additional eight items were therefore drafted to measure feelings relating to life after the birth of a baby and support that might be available to them. In addition to being anxious about how they would cope after the birth of their baby, women with a LTC also expressed concern over how their health can be affected during and after their pregnancy:

*I needed quite a lot of overview from gastroenterology just to make sure that it wasn’t getting worse and what the plan would be if I escalated and I wasn’t going into remission … , it was quite worrying, because I was obviously dealing with all the symptoms of being pregnant which causes issues with your guts anyway, or causes pains everywhere else, and nausea and all that, but I was also dealing with symptoms of my ulcerative colitis which were getting worse. I couldn’t distinguish which one was which most of the time, if it was normal or if it was just my ulcerative colitis, but it was definitely worse, and I needed upping on my meds.* W6, Ulcerative colitis

*… the Well-being in Pregnancy scale, something along the lines of, ‘Are you anxious about how you’ll be able to cope after your pregnancy if you suffer from any kind of long term condition?’ … if somebody’s a parent and they’ve got a long term condition then it’s an absolute guarantee that they worry they’re not being the best parent they can be or they’re concerned about the way that they’re parenting simply because of their condition.* W4, Spinal condition: Diastematomyelia

In light of the views expressed, five items specifically relating to pregnancy and living with a LTC were added to form a standalone WiP module. Finally, in line with participant feedback, response categories for the questionnaire were reduced from a six-point response scale to a four-point response scale which they viewed as a manageable number of response categories and suitable for the item stems.

**Table 1:** Modifications to the WiP questionnaire

Phase 1a	
**Reason for revision**	**Instructions and format**
Participants experienced difficulties differentiating between six response options	Response options reduced from a six-point response scale to a four-point response scale
Preamble added for LTC standalone module	Wording added: *‘A long-term condition (LTC) is any health issue that has lasted, or will last, for at least 12 months. LTCs include memory problems, depression and other mental health conditions as well as physical health conditions such as diabetes and heart disease.* *When answering the following questions, please think about your long-term health condition(s)’*
***Reason for revision***	***Items selected for revision***
Terminology (to reduce risk of socially desirable responses)	Bonded with baby
	Given purpose in life
	Felt supported by the health professionals involved in care
***Reason for revision***	***Items added***
Eight items added to reflect further identified areas of well-being during pregnancy	Enough social contact with other people
	Enjoying pregnancy
	Concerned not enough support from health services after birth
	Confident about caring for baby
	Worried about support after birth
	Prepared for life as a mother
	Concerned about coping when baby is born
	Confident of support from other people after birth
Five items relating to pregnancy and living with a LTC added (stand-alone module)	Able to cope well during pregnancy, despite health condition(s)
	Symptoms of long term health condition(s) bother me during pregnancy
	Confident in managing the day-to-day aspects of health condition(s) during pregnancy
	Able to cope after the birth of baby, despite health condition(s)
	Concerned about managing the day-to-day aspects of health condition(s) after the birth of baby
**Phase 1b**	
No revisions required, two items identified as potentially eliciting socially desirable responses	
**Phase 2**	
***Reason for removal***	***Items selected for deletion***
Floor and ceiling effects	Concerned that relationships with other people important to me are changing
Poor correlations with a large number of items and reduction of Cronbach’s alpha	Concerned about the health of baby
	Concerned about my health during pregnancy
	Pregnancy adds purpose in life
	Physical symptoms of pregnancy upset me
	Anxious about giving birth
Not conceptually integrating with extracted factors and highlighted potentially eliciting socially desirable responses during Phases 1a and 1b	Satisfied with experience of health care during pregnancy
	Overall, supported by the health professionals involved in care during pregnancy

Note: items in table are paraphrased

### Phase 1b: Consultation with patient and public representatives (PPI)

Four women provided feedback on the modified WiP items. The 20 WiP core items and five WiP LTC items were considered relevant and appropriate for use with pregnant women by all PPI participants. No items were deleted and the response categories were thought to be appropriate for the item stems. One item, ‘Overall, I feel supported by the health professionals involved in my care during my pregnancy‘, was thought to be potentially sensitive depending on where the questionnaire was completed. For example, women may feel inclined to provide a positive response if they thought it might affect their care.

### Phase 2: Psychometric Validation

#### Sample Characteristics

In total, 768 pregnant women completed the online survey with 502 (65.4%) women reporting that they did not have a LTC and 266 (34.6%) women reporting that they had one or more LTCs. The average age was 32.1 (SD 4.2) years old with most women remaining in full time education until after they were 19 years old. Most women described themselves as White British (84.9%). For women who reported having one or more LTC, having a mental health condition (46.2%), a joint, bone and connective tissue disorder (29.7%), a respiratory condition (28.6%) or a gastrointestinal condition (20.7%) was the most frequent condition reported. See Table 2 for further details. Regarding experiences of care during pregnancy, 63.1% of women said that they were satisfied with their care during their current pregnancy.

**Table 2:** Participant characteristics

Characteristic	Healthy population ***N*** = 502	LTC population ***N*** = 266	All ***N*** = 768
**Age (years)**			
Mean (SD, range)	32.46 (3.8, 23–45)	31.32 (4.6, 21–42)	32.06 (4.2, 21–45)
**Age when left full time education**			
≤ 16 years old	25 (5.0)	23 (8.6)	48 (6.3)
17–18 years old	84 (16.7)	74 (27.8)	158 (20.6)
≥ 19 years	381 (75.9)	162 (60.9)	543 (70.7)
Still in full time education	6 (1.2)	7 (2.6)	13 (1.7)
Missing	6 (1.2)	–	6 (0.8)
**Partner**			
Yes	487 (97.0)	251 (94.4)	738 (96.1)
No	8 (1.6)	11 (4.1)	19 (2.5)
Missing	7 (1.4)	4 (1.5)	11 (1.4)
**Ethnic group, n (%)**			
White British	416 (82.9)	236 (88.7)	652 (84.9)
White (other)	39 (7.8)	17 (6.4)	56 (7.3)
Black African	1 (0.2)	–	1 (0.1)
Black Caribbean	4 (0.8)	2 (0.8)	6 (0.8)
Asian	17 (3.4)	1 (0.4)	18 (2.3)
Mixed race	14 (2.8)	5 (1.9)	19 (2.5)
Other	3 (0.6)	3 (1.1)	6 (0.8)
Prefer not to say	2 (0.4)	1 (0.4)	3 (0.4)
Missing	6 (1.2)	1 (0.4)	7 (0.9)
**Long-term condition**			
Autoimmune disorder	–	4 (1.5)	4 (0.5)
Cardiovascular condition	–	17 (6.4)	17 (2.2)
Dermatological disorder	–	3 (1.1)	3 (0.4)
Endocrine problem	–	38 (14.3)	38 (4.9)
Gastrointestinal condition	–	55 (20.7)	55 (7.2)
Gynaecological and urinary tract condition	–	14 (5.3)	14 (1.8)
Haematological	–	3 (1.1)	3 (0.4)
Joint, bone and connective tissues disorders	–	79 (29.7)	79 (10.3)
Mental health condition	–	123 (46.2)	123 (16.0)
Neurological conditions including disorders of the peripheral nerves	–	31 (11.7)	31 (4.0)
Other	–	5 (1.9)	5 (0.7)
Respiratory condition		76 (28.6)	76 (9.9)
**Number of long-term conditions**			
1	–	143 (53.6)	143 (18.6)
2	–	80 (30.1)	80 (10.4)
3	–	31 (11.7)	31 (4.0)
4+	–	12 (4.6)	12 (1.6)

#### Item reduction and scale refinement

On examination of item floor and ceiling effects, one item regarding concerns ‘relationships with other people important to me’ changing was removed due to more than 40% of participants responding ‘At no time’. Five of the remaining 19 core WiP items were removed due to having poor correlations with a large number of items and their presence reducing the overall Cronbach’s alpha. Items were iteratively removed, with a new correlation matrix and reliability analysis rerun for each iteration. See Table 1 for further detail.

The KMO value for the remaining 14 items was 0.84, exceeding the recommended value of 0.6. The Bartlett Test of Sphericity reached statistical significance (*P* < 0.01), confirming correlation between the items. Fourteen items were entered into an EFA and four factors explaining 63.32% of the variance were initially extracted. Conceptually, two items regarding satisfaction with health services did not integrate well within the extracted factors and, on consideration of views expressed during interview and PPI feedback regarding such items eliciting socially desirable responses, they were removed from further analysis. Upon their removal, a further EFA determined three factors with an Eigenvalue of greater than one explaining 61.91% of the variance. The three factors were named: 1) Concerns over support after birth, Cronbach’s alpha, 0.76, 2) Positive pregnancy, Cronbach’s alpha, 0.79 and, 3) Confidence about motherhood, Cronbach’s alpha, 0.76. See Table 3 for the factor structure and loadings.

**Table 3:** Structure matrix factor loadings for core WiP items

Item	Factor loading		
	1	2	3
I worry that I will not have enough support after the birth of my baby	**0.845**	0.245	0.429
I am concerned I will not have enough support from health services after the birth of my baby	**0.800**	0.186	0.214
I feel confident I will be supported by other people after the birth of my baby	**0.718**	0.286	0.415
I have enough social contact with other people	**0.623**	0.308	0.132
I am enjoying my pregnancy	0.285	**0.832**	0.233
I feel very positive about being pregnant	0.241	**0.769**	0.243
Being pregnant makes me feel confident	0.284	**0.748**	0.194
I feel I connected to my baby	0.160	**0.730**	0.396
I am happy with how I look in pregnancy	0.246	**0.709**	0.091
I feel confident about caring for my baby	0.320	0.248	**0.886**
I feel prepared for life as a mother	0.275	0.287	**0.879**
I am concerned about how I will cope when my baby is born	0.516	0.295	**0.676**

Rotation method: Oblimin with Kaiser Normalization

The five WIP-LTC module items were entered into a separate EFA which identified a single five item factor explaining 59.9% of the variance which supported its use as a single scale. Reliability, as assessed with Cronbach’s alpha, was 0.83. See Table 4 for the factor loadings.

**Table 4:** Factor loadings for WiP LTC stand-alone module

Item	Factor loading
	1
I am able to cope well during pregnancy, despite my health condition(s)	0.843
I feel confident in managing the day-to-day aspects of my health condition(s) during my pregnancy	0.805
I feel that I will be able to cope after the birth of my baby, despite my health condition(s)	0.743
Symptoms of my long term health condition(s) bother me during my pregnancy	0.738
I am concerned about managing the day-to-day aspects of my health condition(s) after the birth of my baby	0.734

#### Scale Distributions and Validation

Each scale was transformed to a 0–100 metric, where 0 indicated low levels of well-being and 100 indicated high levels of well-being. Scale scores were calculated by summing the response values, dividing the summed score by the maximum raw score and multiplying by 100. No scale exhibited floor or ceiling effects, which was considered to be > 20% of responses achieving the minimum or maximum score. Minimal respondents achieved scores of 0 on scales ‘Concerns over support after birth’, ‘Positive pregnancy’ and on the LTC module, while 8.9% of respondents achieved the maximum score on scale ‘Confidence about motherhood’. The WiP Total score displayed no floor or ceiling effects. Scale statistics are reported in Table 5.

**Table 5:** Scale score descriptive statistics

Measure	Mean (SD), Range		
	Healthy population ***N*** = 502	LTC population ***N*** = 266	All ***N*** = 768
**WiP**			
Factor 1: Concerns over support after birth	50.3 (21.8), 0–100	49.31 (22.0), 0–100	50.0 (21.9), 0–100
Factor 2: Positive pregnancy	52.5 (19.0), 0–100	45.7 (21.7), 0–100	48.7, (20.2), 0–100
Factor 3: Confidence about motherhood	61.0 (20.7), 0–100	64.3 (24.2), 0–100	62.1 (22.0), 0–100
WIP Total score	54.59 (16.0) 7.22–96.30	53.11 (17.6) 2.22–97.22	54.08 (16.6) 2.22–97.22
WIP: LTC module	–	55.4 (20.3), 6.7–100	–
**EQ-5D-5L**			
	0.73 (0.17), −0.33 - 1.0	0.61 (0.23), − 0.12 - 1	0.69 (0.20), − 0.33 - 1.0
**LTCQ**			
	–	57.4 (17.4), 20–100	–
**WHO5**			
Total score	41.2, (17.5), 0–96	–	–
**SWLS**			
Total score	24.6 (6.0), 5–35	–	–
**PANAS**			
Positive affect	26.0 (7.1), 10–47	–	–
Negative affect	22.9 (7.6), 10–50	–	–

Relationships between the WiP scales and potential covariate factors were examined. No significant differences between the healthy group and LTC group was found for scale ‘Concerns over support after birth’ and for scale ‘Confidence about motherhood’. Significant differences were found between the healthy group and LTC group for scale ‘Positive pregnancy’. Healthy women reported experiencing a more positive pregnancy compared to women with one or more LTC; mean (SD) = 52.5 (19.0) v 45.7 (21.7), t_481.73_ = − 4.251, *p* < 0.001. Differences between groups were also shown on the EQ-5D-5L where healthy women reported a higher QoL than those living with one or more LTC; mean (SD) = 0.73 (0.17) v 0.61 (0.23), t_423.5_ = − 7.47, *p* < 0.001. No significant differences were identified for any WiP scale when comparing women according to the number of reported LTCs (ANOVA, all comparisons *p* > 0.05).

While there was no significant difference for scale ‘Concerns over support after birth’, ‘Confidence about motherhood’ and the LTC module, those who reported having a planned pregnancy reported significantly higher levels of ‘Positive pregnancy’; means = 51.9 v 42.45, t_766_ = 5.13, *p* < 0.001.

As expected, for the healthy population group, relationships between WiP scales and the WHO-5, SWLS and the PANAS positive and negative affect scales were moderate (see Table 6). Relationships with the EQ-5D-5L were weak for ‘Concerns over support after birth’ (*r* = 0.25, *p* < 0.01) and ‘Confidence about motherhood’ (*r* = 0.26), but moderate for ‘Positive pregnancy’ (*r* = 0.38). For the LTC cohort, correlations were moderate to high with the EQ-5D-5L and LTCQ, *r* = 0.52, *p* < 0.01 and *r* = 0.70, *p* < 0.01 respectively, indicating the scales were measuring similar but different concepts. See Table 6 for correlations between well-being measures.

**Table 6:** Correlations between all well-being measures

WIP Factor	WHO-5*	SWLS*	PANAS Positive*	PANAS negative*	EQ-5D	LTCQ**
**1: Concerns over support after birth**	0.405	0.334	0.370	−0.438	0.246	0.294
**2: Positive pregnancy**	0.572	0.397	0.576	−0.449	0.384	0.467
**3: Confidence about motherhood**	0.293	0.345	0.335	−0.334	0.149	0.257
**WiP total**	0.537	0.458	0.542	−0.521	0.331	0.432
**WiP_LTC**	–	–	–	–	0.524	0.704

All significant *p* < 0.01, *Health population only, **LTC population only

## Discussion

The WiP was initially developed based on existing literature and feedback from women who were pregnant or had recently given birth. This paper reports on steps taken to further refine, modify and validate WiP core items to assess well-being in pregnancy and to provide an additional bolt on LTC module to assess well-being among women living with one or more LTC. Phase 1 used cognitive interviews and PPI engagement to gain feedback from women and healthcare professionals regarding the understanding of the items. Phase 2 administered an online survey to help confirm the presence of three sub-scales and an additional stand-alone LTC module.

The content validity of the revised questionnaire is strengthened through further input by pregnant women and women who had recently given birth ensuring greater awareness of sensitive questions allowing women to communicate concerns more freely. Additional items regarding support following birth ensures a greater breath in the measurement of well-being. Revised scales demonstrated good validity and internal reliability. The first factor, ‘Concerns over support after birth’ contains four items asking women about worries or concerns they have regarding adequate support and social contact. The second factor, ‘Positive pregnancy’ contains five items measuring positive feelings during pregnancy. Positive feelings in pregnancy where shown to be significantly higher in the healthy women cohort compared to women with one or more LTC, and higher in women who had planned their pregnancy. The third factor, ‘Confidence about motherhood’ contains three items asking women how they feel about life and coping as a mother. Scores for the three scales identified moderately correlated with the included, established well-being measures indicating that they were measuring similar, yet distinct concepts. The LTC stand-alone module contained five items which all loaded highly on one factor and correlated moderate to highly with the EQ-5D-5L and the LTCQ. The items of the revised WiP and LTC bolt on were positively appraised by women and health professionals.

As noted earlier, well-being measures, such as the WiP, that can identify positive mental health rather than simply the absence of illness, should find a natural home in maternity care. The lack of uptake of these measures may be related, at least in part, to the need to raise awareness of maternal mental health difficulties. It is widely recognised that mental health problems during the perinatal period are more prevalent and can frequently go unrecognised and untreated, with some women not seeking help because of fear of stigma, or fear of intervention by social services [40]. Guidance has been developed to improve the identification of women with mental health problems with short screening tools. A shift in focus to well-being measures may be seen to distract and detract from the needs of women who have mental health difficulties. However they should be complementary. Well-being measures and models in pregnancy may go some way to help reduce the stigma associated with the negative focus of existing measures.

A growing interest within general psychiatry to promote well-being models may also help facilitate the use of well-being measures in policy and practice more broadly. Fava and GUidi (2020) argue there is a role in psychiatry for promoting euthymia (a state of internal calm and contentment) and supporting positive emotions, meaning and purpose, competence, achievements, and quality relationships as many conditions are chronic and prone to relapse after treatment [41]. Macleod (2020) identifies potential benefits in how we conceptualise mental illness and how we treat it, and that it is after treatment that more well-being focused approaches can come into their own, by both reducing residual symptoms and building well-being resource [42].

Psychometric research has demonstrated considerable overlap between well-being measures and measures of common mental disorders [43] and studies have demonstrated a strong negative correlation between measures of depression and subjective well-being around the time of birth [44]. Much more methodological research is needed to help us understand the uniqueness and commonalities of the measures we use and to maximise their ability to identify women with mental health difficulties while highlighting the full spectrum of psychological well-being.

Paramount in considering the best measures to use in research, policy and practice is what women think about the measures we use. Reframing how we conceptualise mental health to include well-being measures should be inclusive and supportive of all women. Phase 1 of this study highlights the acceptability of the WiP with women and practitioners and consideration should be given to how such measures are used in practice. Pregnancy specific well-being measures provide a route into asking women in more detail about how their care may be tailored to support them were needed and also facilitates positive conversations with women about how care and experience of pregnancy and childbirth may be enhanced further.

Whilst this study benefited from a large sample size which spanned a considerable range of physical and mental LTCs, the majority of participants for the online survey were recruited via social media which may impact the generalisability of the findings. Social media advertisements however enabled women of child bearing age within the UK to be effectively targeted. In addition, social media is becoming an increasingly established method of recruitment for pregnancy and infant health research, particularly for studies requiring online survey completion [45]. Although known to be less effective in recruiting pregnant women than social media advertisements [46], traditional recruitments approaches were also adopted through advertisements posted on relevant mother and baby and condition specific organisations websites allowing women who did not use social media the opportunity to take part.

It is noteworthy that just under half of the women living with a pre-existing LTC who completed the online survey reported living with a mental health condition. This is in contrast to those taking part in the cognitive interviews where only one of the 11 women reported a pre-existing LTC. Many of these women also reported living with a physical LTC, however, the modified WiP may benefit from further conceptual testing among women living with a mental health condition.

Finally, we acknowledge women who had recently given birth were included in Phase 1 cognitive interviews. Whilst the WiP is a measure intended for women who are currently pregnant, those who had recently given birth were able to give an important insight into the full pregnancy journey, ensuring all stages of pregnancy were considered.

## Conclusion

Subjective well-being measures, which capture both positive and negative emotions, are important tools when assessing mental health for all women during pregnancy and childbirth. This study highlights the acceptability of the WiP with women and healthcare professionals. After a period of item refinement, analyses confirmed good psychometric properties of the WiP questionnaire. The use of pregnancy specific well-being measures provide a route into asking women in more detail about how their care may be tailored to support them and can facilitate positive conversations with women about how care and experience of pregnancy and childbirth may be enhanced further. Their use also has the potential to reduce fragmented care through promoting multidisciplinary discussions between healthcare professionals along the course of a woman’s pregnancy.

## Supplementary Information

**Additional file 1.** Refinement and development of the Well-being in Pregnancy (WiP) questionnaire interview topic guides. File included women living with LTCs and healthcare professional’s topic guides for Phase 1.

## Data Availability

The datasets generated and analysed during the current study are not publicly available due to ethical concerns regarding the possibility to fully anonymise data but are available from the corresponding author on reasonable request.

## Abbreviations

EFA: Exploratory factor analysis
KMO: Kaiser-Meyer-Olkin
LTC: Long-term condition
LTCQ: Long-Term Conditions Questionnaire
NHS: National Health Service
PANAS: Positive and Negative Affect Schedule
PPI: Patient and Public Involvement
r: Pearson correlation coefficient
SD: Standard deviation
SWLS: Satisfaction With Life Scale
UK: United Kingdom
VAS: Visual Analogue Scale
WHO-5: WHO-5 Well-being Questionnaire
WiP: Well-being in Pregnancy Questionnaire
