# Correction: Refinement of the Well-being in Pregnancy (WiP) questionnaire: cognitive interviews with women and healthcare professionals and a validation survey

**DOI:** 10.1186/s12884-022-04730-y

**Published:** 2022-05-12

**Authors:** Laura Kelly, Jennifer J. Kurinczuk, Ray Fitzpatrick, Fiona Alderdice

**Affiliations:** 1 Health Services Research Unit, Nuffield Department of Population Health, University of Oxford, Oxford, UK; 2 Harris Manchester College, University of Oxford, Oxford, UK; 3 NIHR Policy Research Unit Maternal and Neonatal Health and Care, National Perinatal Epidemiology Unit, Nuffield Department of Population Health, University of Oxford, Richard Doll Building, Old Road Campus, Headington, Oxford, OX3 7LF UK

**Correction: BMC Pregnancy Childbirth 22, 325 (2022)**

**https://doi.org/10.1186/s12884-022-04626-x**

Following publication of the original article [1], the author reported that the 2nd author “Research Officer” should be deleted from the author group. Research Officer was incorrectly added to the author list.

The original article [1] has been corrected.
