# Supplementary material for: Refinement of the Well-being in Pregnancy (WiP) questionnaire: cognitive interviews with women and healthcare professionals and a validation survey
Source: BMC Pregnancy Childbirth. 2022 Apr 15;22:325. doi: 10.1186/s12884-022-04626-x (PMC9013132; doi:10.1186/s12884-022-04626-x)
Supplement: Supplementary file 1 — Additional file 1. Refinement and development of the Well-being in Pregnancy (WiP) questionnaire interview topic guides. File included women living with LTCs and healthcare professional’s topic guides for Phase 1. [file 12884_2022_4626_MOESM1_ESM.docx]

**Refinement and development of the Well-being in Pregnancy (WiP) questionnaire interview topic guides**

Dr Laura Kelly, Research Officer^1, 2^

Professor Jennifer J Kurinczuk^3^

Professor Ray Fitzpatrick^1^

Professor Fiona Alderdice^2, 3^

^1^Health Services Research Unit, Nuffield Department of Population Health, University of Oxford

^2^Harris Manchester College, University of Oxford

^3^National Perinatal Epidemiology Unit, Nuffield Department of Population Health, University of Oxford

Corresponding author: Fiona Alderdice, National Perinatal Epidemiology Unit, Nuffield Department of Population Health, University of Oxford, Richard Doll Building, Old Road Campus, Headington, Oxford, OX3 7LF. Email: Fiona.alderdice@ndph.ox.ac.uk

**Women living with LTCs topic guide**

1. Can you start by telling me a little bit of background about yourself?
2. Can you tell me about any long-term health conditions you have?
3. Can you tell me about any impacts your health had on daily life before pregnancy?
4. Can you tell me about any impacts your health had on daily life during/after pregnancy?
5. Can you tell me about your current antenatal or postnatal care?
6. [Repeat for each exemplar measure] Can you tell me your initial thoughts or impressions of the questions in the [insert measure] and how relevant they may be for you?

- Probe around preamble and relevance of the items
- Format and layout
- Concerns regarding any of the items
- Anything not covered which you would expect to be included in this questionnaire?

1. Having looked at each of the questionnaires, it would be good to get your thoughts on whether you think the use of these questionnaires would be helpful:

- When you see healthcare professionals?
- For research purposes?

**Healthcare professional’s topic guide**

1. Can you start by telling me a little bit about your current role and background, particularly in regards to caring for: 1) Pregnant women, 2) Postpartum women, 3) women living with LTC(s)?
2. Do you currently use, or have any experience of using, health and wellbeing outcome or experience measures in clinical practice or research?
3. [Repeat for each exemplar measure] Can you tell me your views on the questions within the [exemplar measure] and how relevant they might be for any women you care for?

- Probe around relevance of the items and the preamble for use with pregnant/postpartum women with LTC(s)
- Is anything not covered which you would expect to be asked in this questionnaire?
- Do you have any concerns regarding any of the items or topics?

1. Having looked at each of the questionnaires, do you think the use of these (or any other) questionnaires would be beneficial: 1) For use with a pregnant woman who has one or more LTC? , 2) For use with a postpartum woman who has one or more LTC?

- Probe around contexts: care planning, research, audits
- Probe around implementation, administration, potential challenges
